# Supplementary material for: Hydrogen-bonding and π-π interaction promoted solution-processable covalent organic frameworks
Source: Nat Commun. 2023 Dec 11;14:8181. doi: 10.1038/s41467-023-43905-9 (PMC10713689; doi:10.1038/s41467-023-43905-9)
Supplement: Supplementary file 1 — Supplementary Information [file 41467_2023_43905_MOESM1_ESM.pdf]

Supplementary Information

Hydrogen-bonding and "π-π" interaction promoted solution-processable covalent organic frameworks

Lei Zhang[1,2], Qiu-Hong Zhu,[1] Yue-Ru Zhou,[1] Shuang-Long Wang,[1] Jie Fu,[1] Jia-Ying Liu,[1] Guo-Hao Zhang,[1] Lijian Ma,[1] Guohua Tao*,[2] Guo-Hong Tao*,[1] Ling He*[1]

[1] Dr. L. Zhang, Q.-H. Zhu, Y.-R. Zhou, S.-L. Wang, G.-H. Zhang, Prof. L. Ma, Prof. G.-H. T, Prof. L. He.
College of Chemistry, Sichuan University, Chengdu 610064, China
E-mail: taogh@scu.edu.cn, lhe@scu.edu.cn
[2] Dr. L. Zhang, Prof. G. Tao
School of Advanced Materials, Peking University Shenzhen Graduate School, Shenzhen 518055, China
E-mail: taogh@pkusz.edu.cn

Table of Contents

1 Supplementary Notes
2 Supplementary Methods
2 Supplementary Methods
3 Supplementary Notes
4 Supplementary Reference

Supplementary Notes

All chemical reagents and solvents were commercially available and used without further purification unless otherwise indicated. Ultrapure water of 18 MΩ cm was used throughout the experiments. Benzidine (98%) was purchased from J&K Scientific Ltd. (China). Hydrazine hydrate (98%) and hexamethylenetetramine (99%) were purchased from Sinopharm Chemical Reagent Co., Ltd. Phloroglucinol (98%), *p*-phenylenediamine (98%), trifluoroacetic acid (99%), 1,4-dioxane (>99.9), and mesitylene were purchased from Energy Chemical.

Supplementary Methods

Infrared spectra (IR) were obtained from Bruker ALPHA infrared spectrometer. Solid-state cross polarization magic angle spinning (CP/MAS) ^{13}C nuclear magnetic resonance spectrum was conducted on an Agilent NMR-vnmrs600 NMR spectrometer. ^{1}H NMR spectra were taken on Bruker 400 MHz nuclear magnetic resonance spectrometer with deuterium oxide (D_2O) or deuterated chloroform ($CDCl_3$) as the locking solvent. Elemental analyses (H, C, N) were performed on an Elementar Vario MICRO CUBE elemental analyzer. Powder X-ray diffraction (PXRD) patterns were recorded by Bruker D8 Advance diffractometer using Cu K_α radiation at 40 kV, 40 mA power. Scanning electron microscopy (SEM) characterization was conducted using a HITACHI SU8010 scanning electron microscope equipped with a field emission gun (FEG) source, but before that, a preliminary look at the material was made using Hitachi TM3000 scanning electron microscope. The nitrogen adsorption and desorption were measured by Micromeritics ASAP 2460 surface area & porosimetry system. The samples were activated at 393 K for 12 h under a vacuum before measurement. The pore size distribution is calculated with Density Functional Theory (DFT) using Innovative MicroActive Software. Dynamic light scattering measurements were conducted on a Malvern Zetasizer Nano-ZS particle size analyzers.

Supplementary Methods

Synthesis of 1,3,5-triformylphloroglucinol. The 1,3,5-triformylphloroglucinol is synthesized according to the published procedure.[1] To a mixture of phloroglucinol (3.0 g, 23.8 mmol) and hexamethylenetetramine (7.4 g, 52.5 mmol), trifluoroacetic acid (50 ml) was added. The solution was heated at 100 ºC for 2.5 h under nitrogen atmosphere. Then, added 150 ml HCl (3 M) and heat at 100 ºC for 1 h. After cooling to room temperature, the reaction mixture was filtered. The filtrate obtained was extracted with dichloromethane (3 × 100 ml), dried over anhydrous Na_2SO_4 and $MgSO_4$. The solution was concentrated to obtain orange solid (23%). IR: ν_{max} in cm^{-1} 1639, 1597, 1431, 1393, 1251, 1192, 965, 872, 818, 785, and 604. ^{1}H NMR (400 MHz, $CDCl_3$): δ 14.12 (s, 3H), 10.15 (s, 3H).

Supplementary Notes

Supplementary Fig.1 Pore size distribution of TpHa COF.

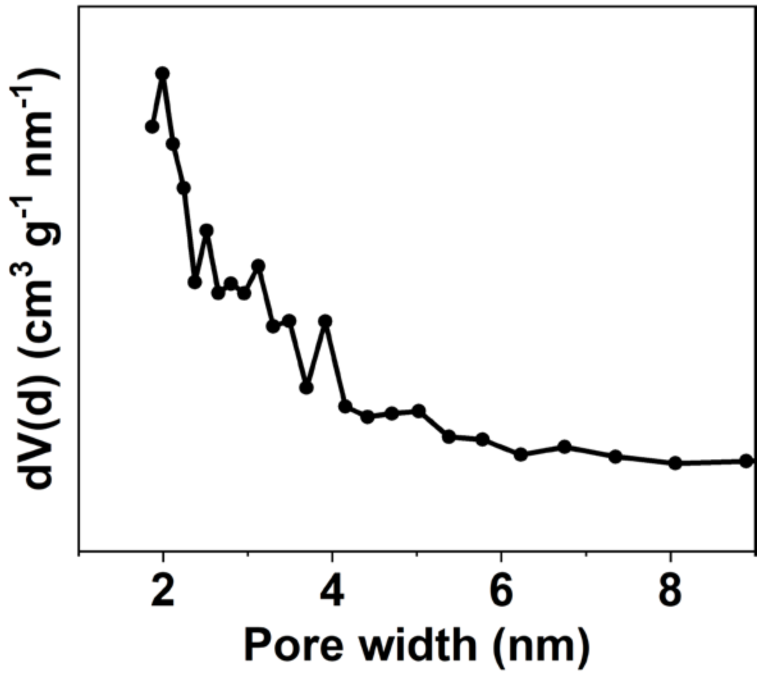

Supplementary Fig.2 Pore size distribution of TbPa COF.

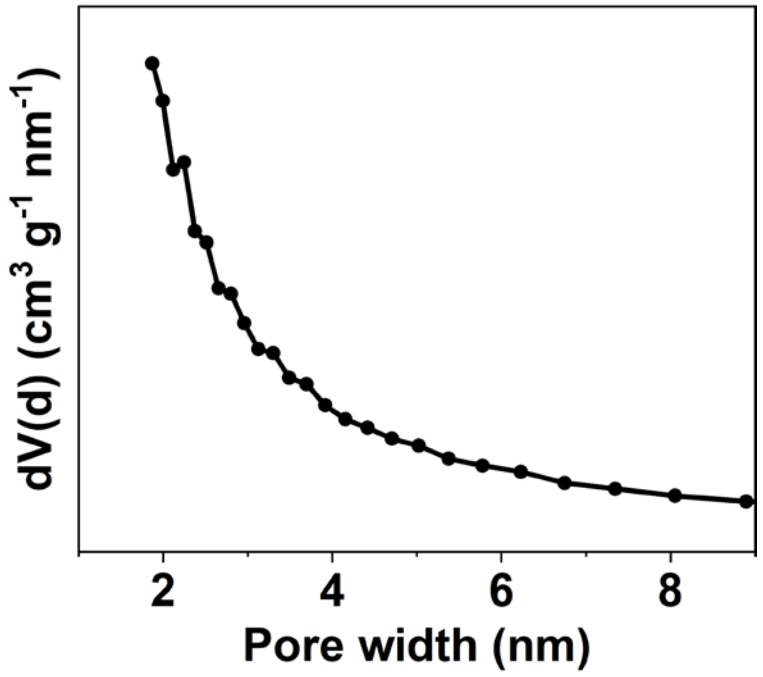

Supplementary Fig.3 Pore size distribution of **TpBd** COF.

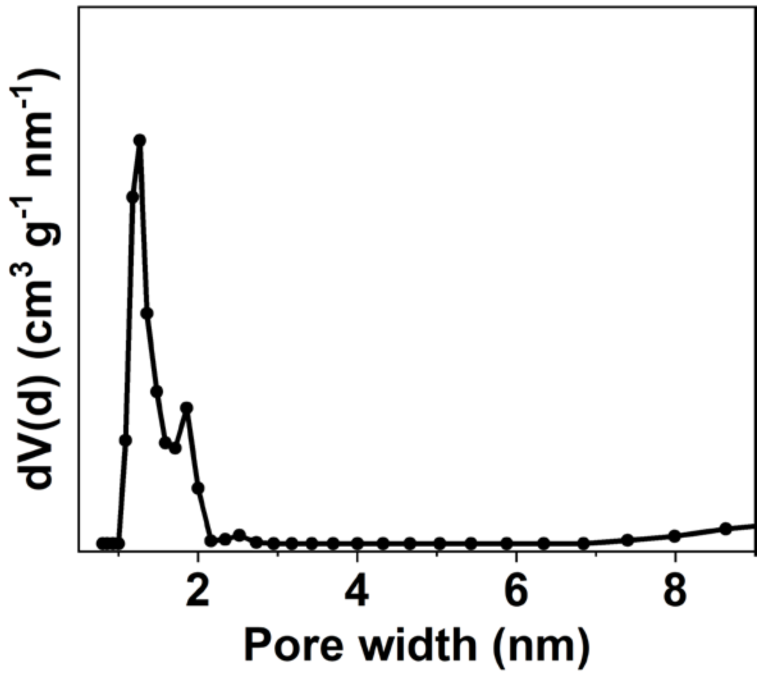

Supplementary Fig.4 Photoimages of the dispersion of TpBd COF in ethanol, dichloromethane, acetone, and water.

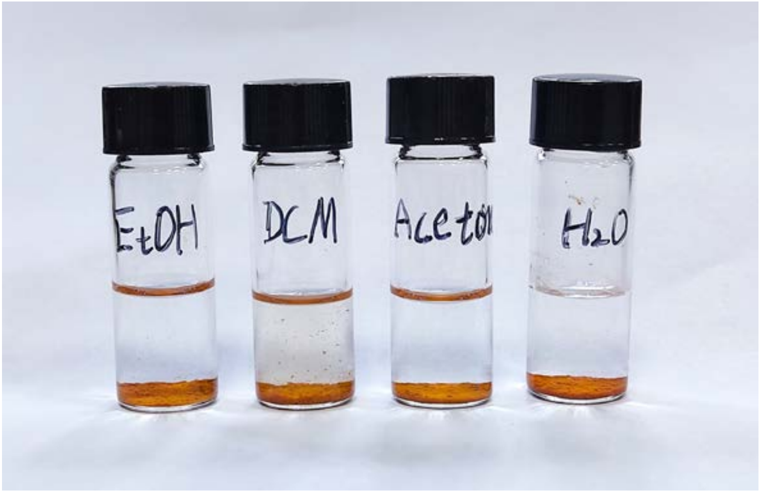

Supplementary Fig.5 NMR spectrum of recycled [C_8mim][Br] in D_2O.

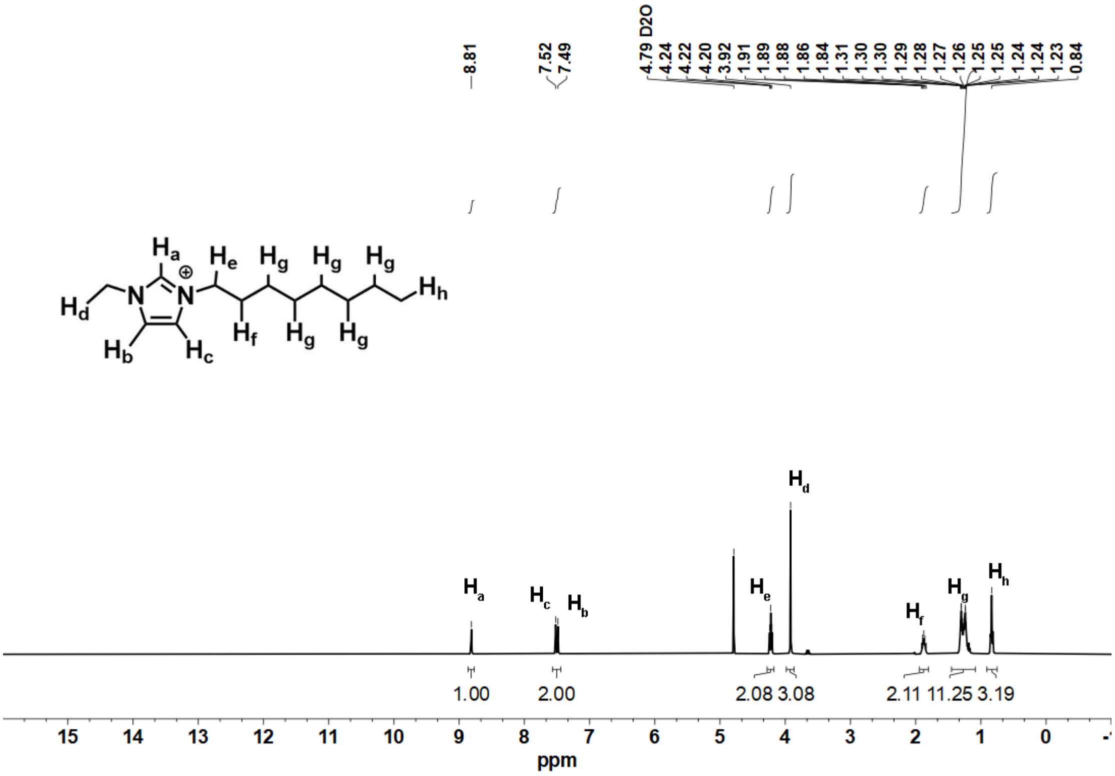

Supplementary Fig.6 Photoimages of TpBd COF in [C_8mim][Br] and ethanol mixture solution after centrifugation.

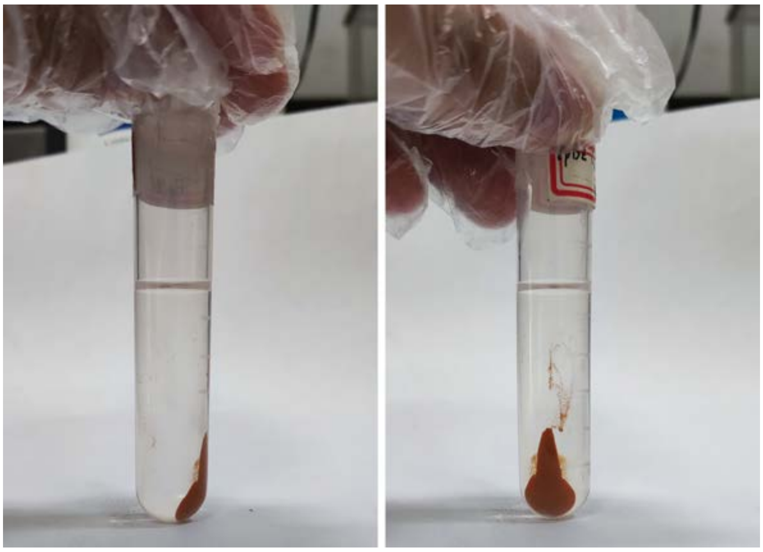

Supplementary Fig.7 FTIR spectra of pristine and treated COFs.

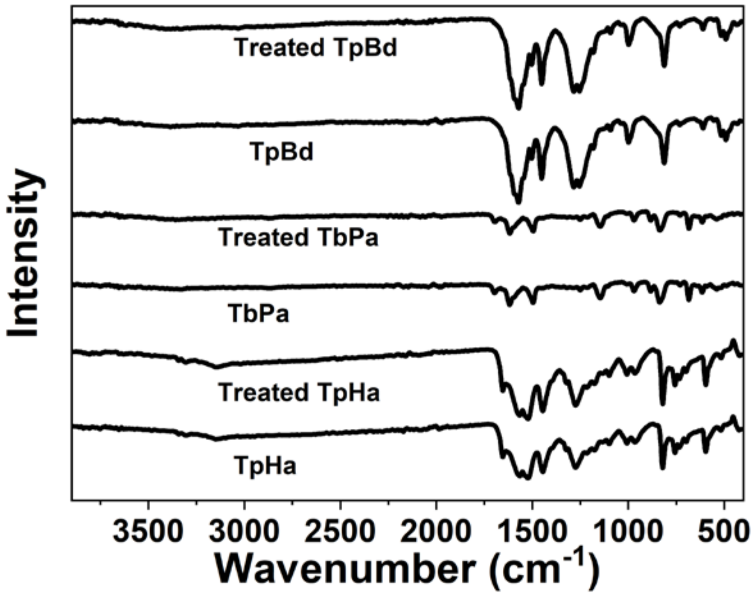

Supplementary Fig.8 XRD pattern of pristine and treated COFs.

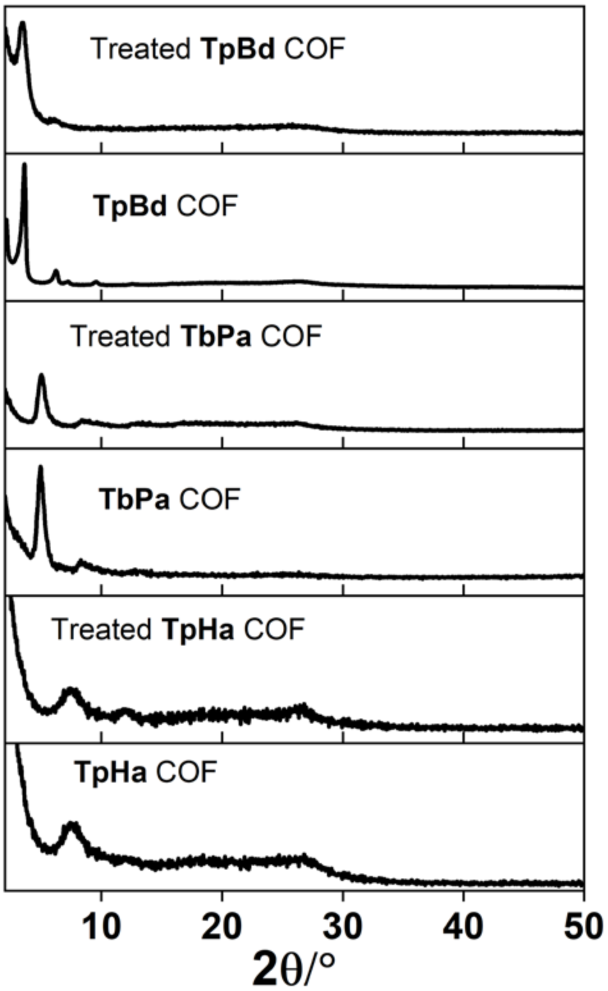

Supplementary Fig.9 Nitrogen-adsorption isotherm curves measured at 77 K for treated TpHa, TbPa, and TpBd COFs, adsorption and desorption data points are represented by filled and empty symbols, respectively.

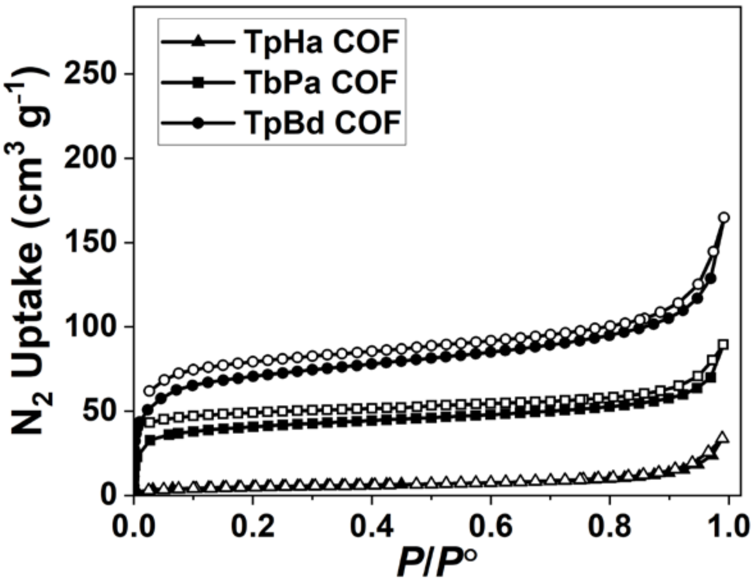

Supplementary Fig.10 Pore size distribution of treated TpHa COF.

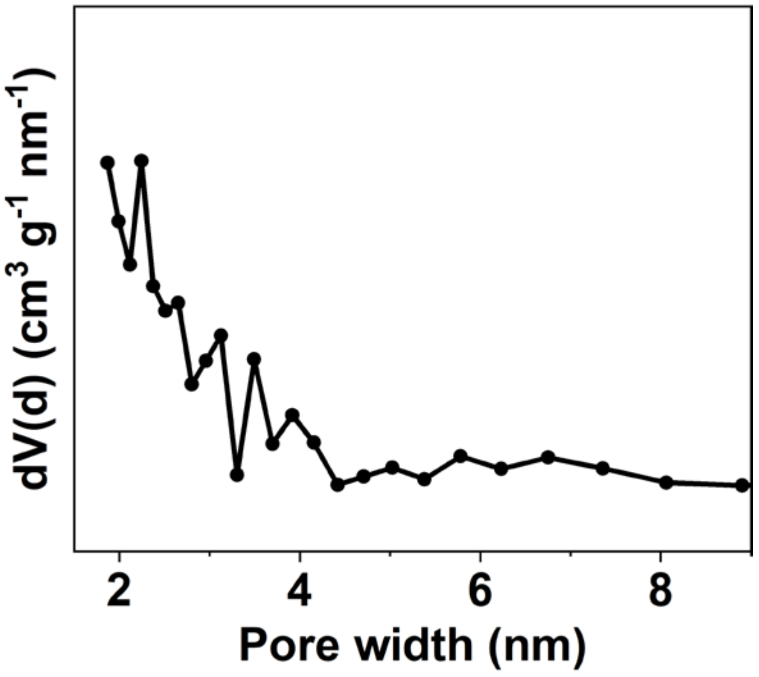

Supplementary Fig.11 Pore size distribution of treated TbPa COF.

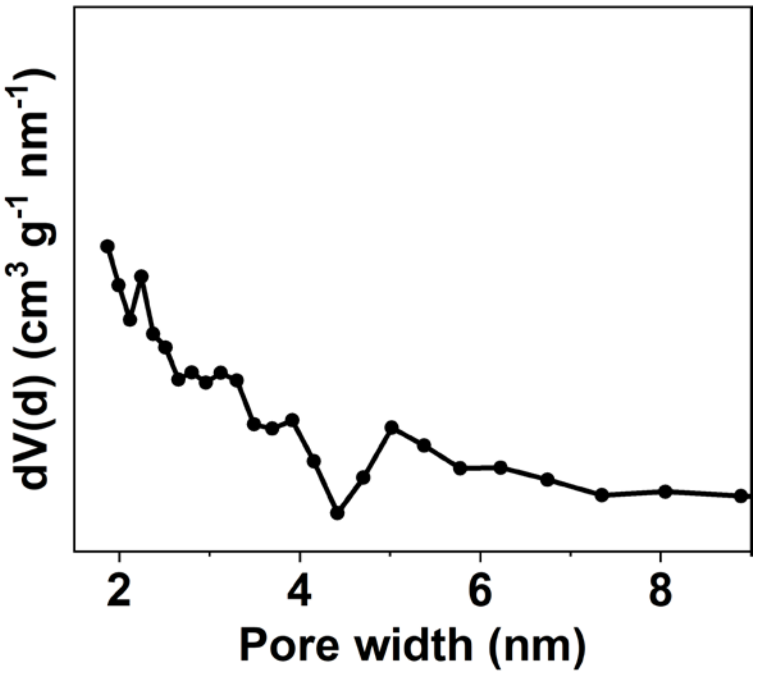

Supplementary Fig.12 Pore size distribution of treated TpBd COF.

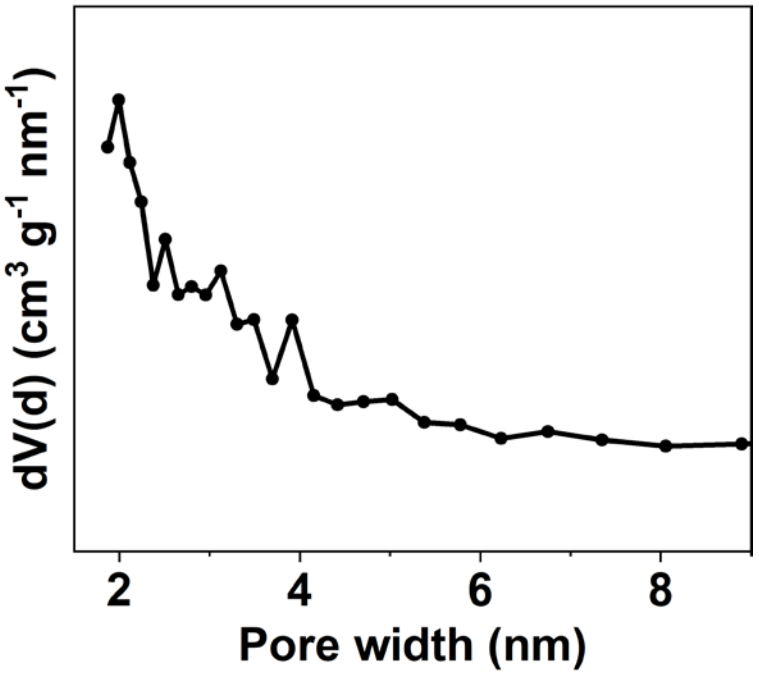

Supplementary Fig. 13. DLS particle size distribution profiles of treated TpBd COF particles in protic and non-protic solvents.

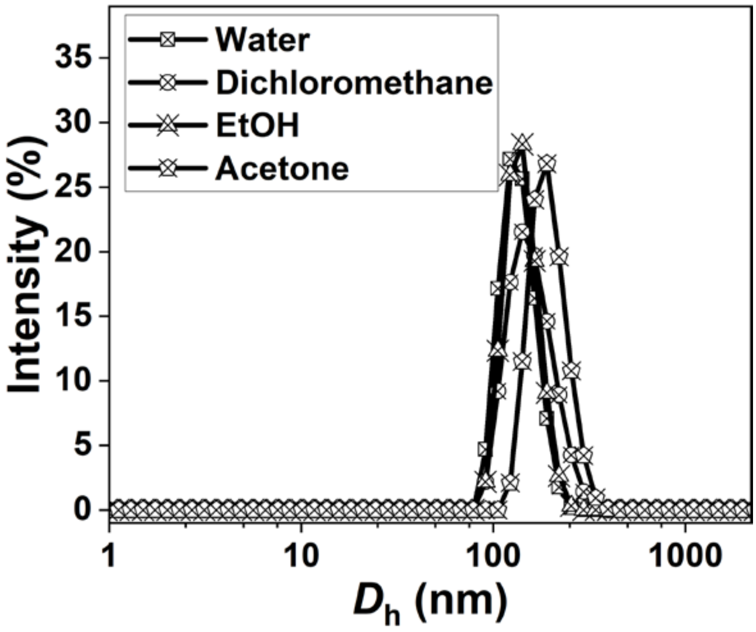

Supplementary Fig. 14. Photoimages of TpBd COF particles in ethanol with different aging times.

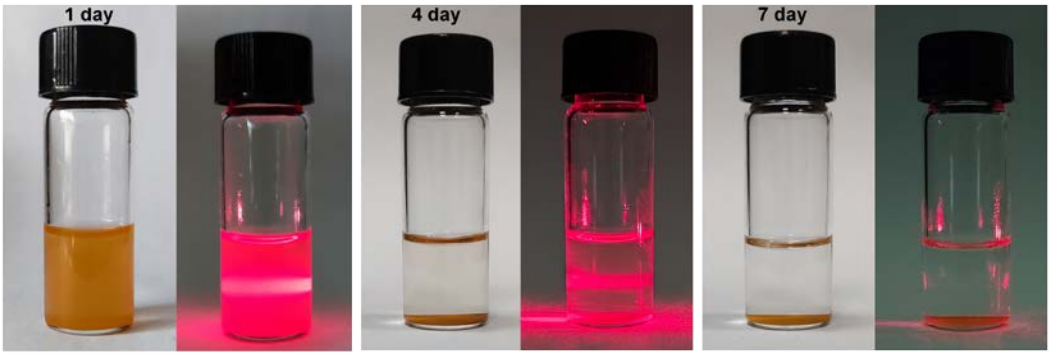

Supplementary Fig.15 Iodine vapor uptake over time by pristine and treated TpBd COFs at ambient pressure and 77 ºC.

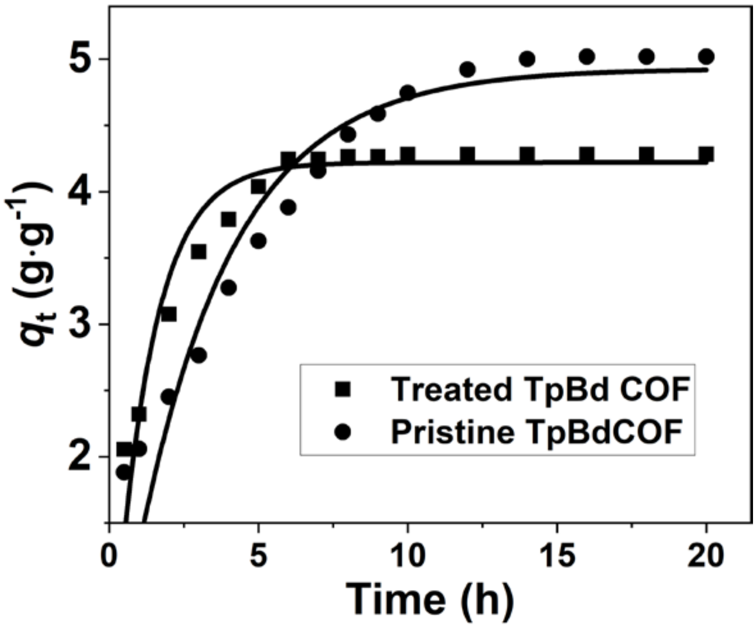

Supplementary Fig.16 Center-of-mass SDF of anions (blue) and cations (red) around the TpBd COF.

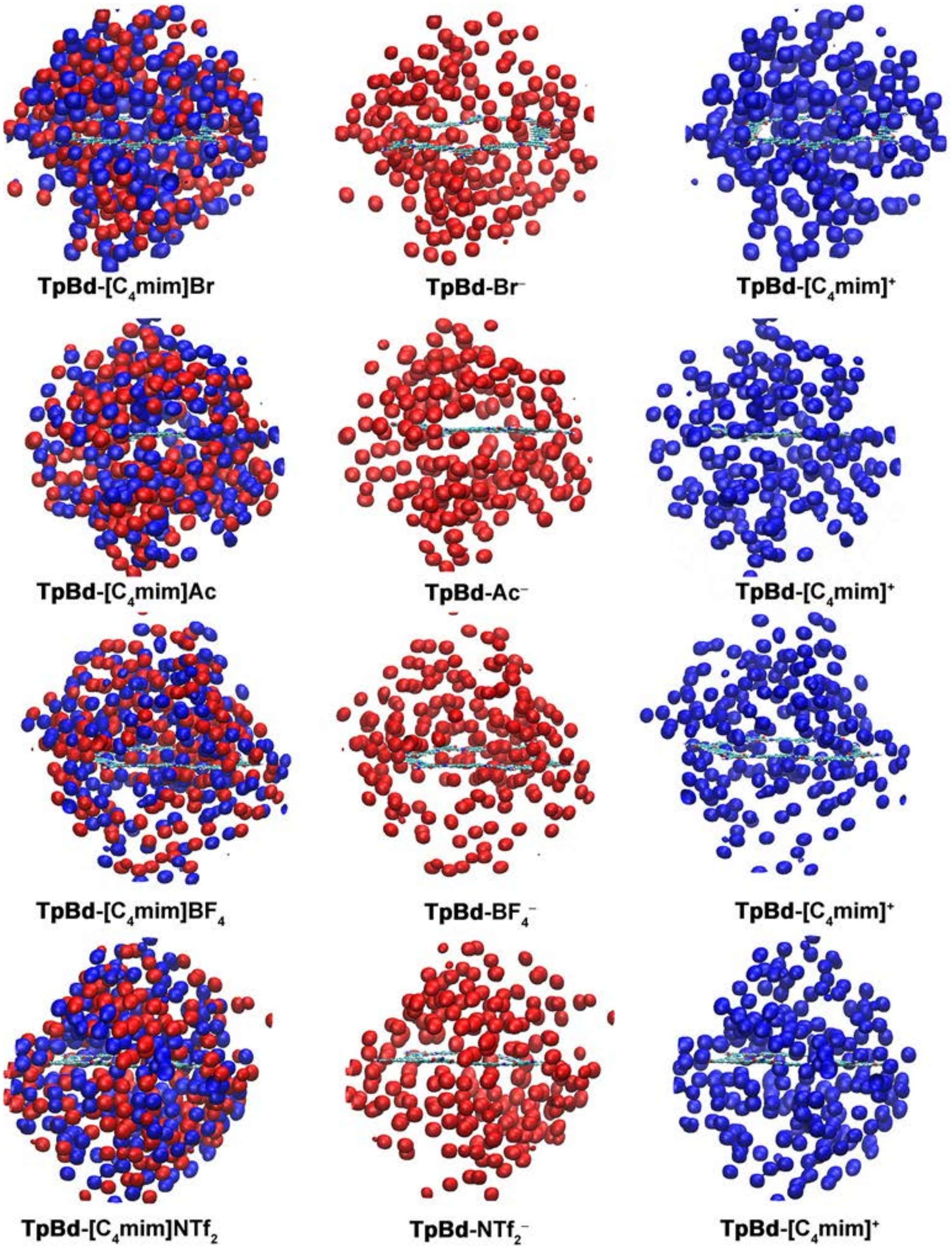

Supplementary Fig.17 Center-of-mass SDF of anions (blue) and cations (red) around the TpBd COF.

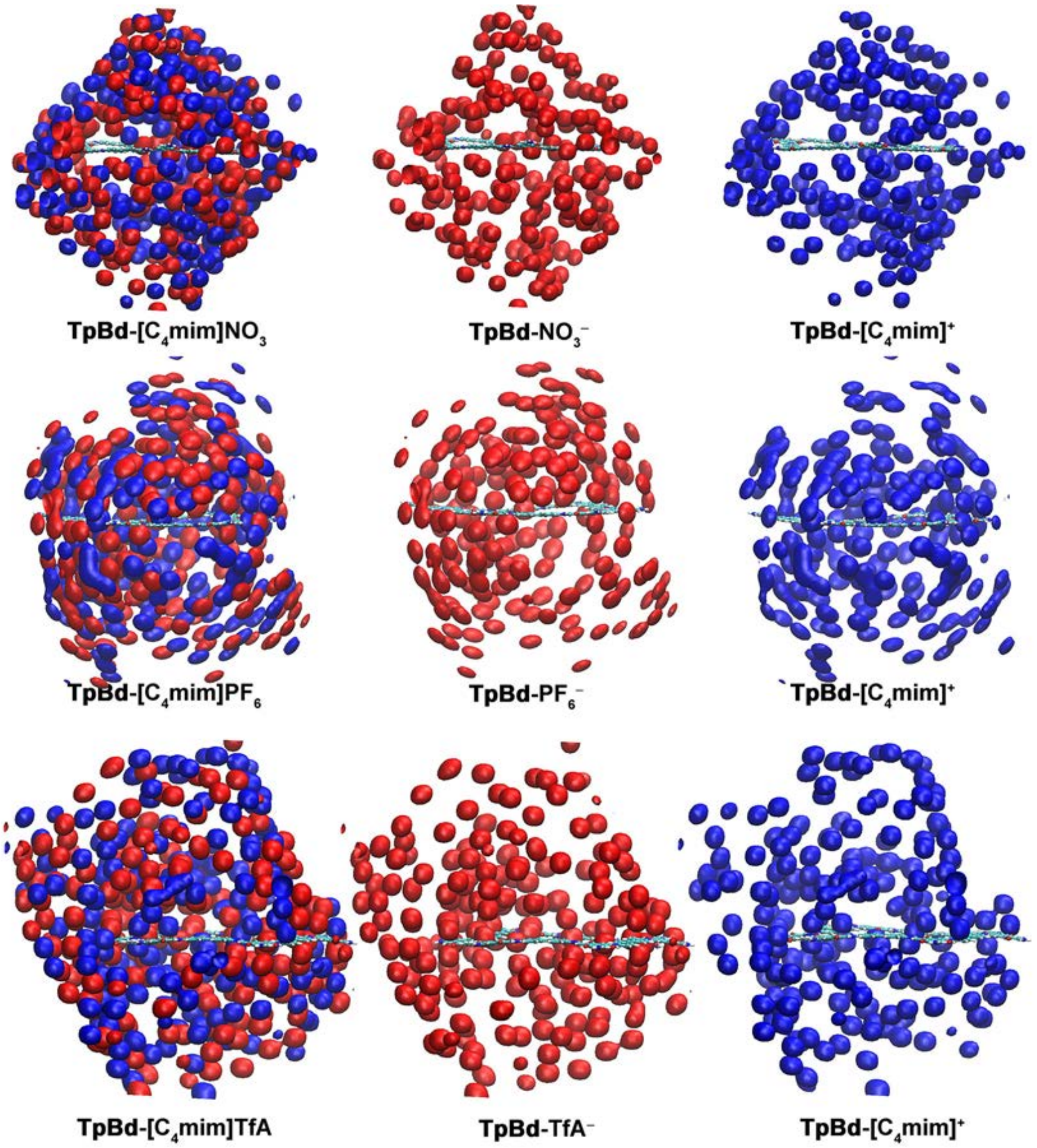

Supplementary Fig.18 Electrostatic potential (ESP) on molecular van der Waals (vdW) surface of anions.

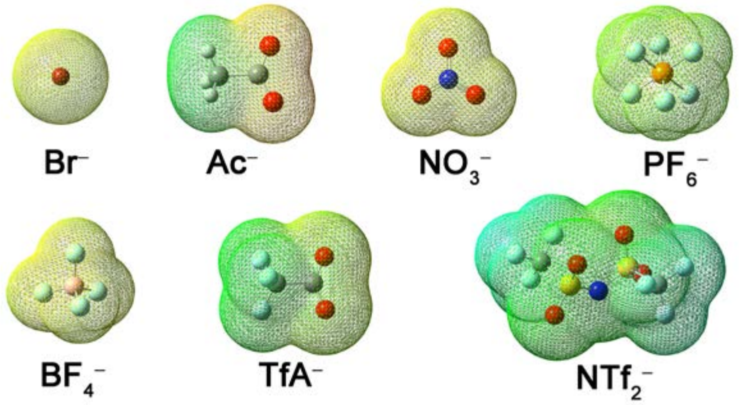

Supplementary Fig.19 Typical TpBd COF and [C_4mim] complexes extracted from the MD simulations. The distances are in angstroms (Å).

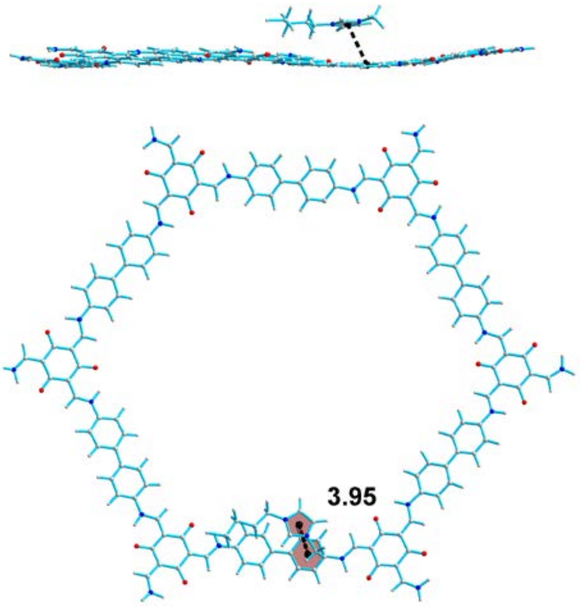

Supplementary Table 1 Elemental analysis for pristine and treated COFs

	C	H	N
Pristine TpHa	51.6	4.1	23.2
Treated TpHa	51.5	4.1	23.2
Pristine TbPa	78.9	4.1	15.0
Treated TbPa	77.9	4.8	14.9
Pristine TpBd	74.9	4.6	9.4
Treated TpBd	74.2	4.8	9.2

Supplementary Table 2 Properties of anions used in this paper.

	Volume ($Bohr^3$)	Surface area ($Bohr^2$)	Min. E[1] (kcal/mol)	Max.E[2] (kcal/mol)	⊿E[3] (kcal/mol)
**Br^-**	346.4	238.9	-142.4	-142.3	0.1
**Ac^-**	489.0	325.7	-166.4	-78.9	87.1
**NO_3^-**	339.5	254.7	-156.7	-122.5	34.2
**TfA^-**	622.1	392.1	-142.7	-88.2	54.5
**BF_4^-**	378.8	272.7	-141.6	-129.6	12.0
**PF_6^-**	521.0	349.0	-124.4	-114.0	10.4
**NTf_2^-**	1368.4	717.4	-117.3	-51.2	66.1

[1] Minimal surface electrostatic potential; [2] Maximal surface electrostatic potential; [3] Difference between minimal and maximal surface electrostatic potential.

Supplementary Table 3. RESP charge of TpBd COF model compound.

atom	charge	atom	charge	atom	charge	atom	charge	atom	charge
H1	0.109209	O51	-0.54866	C101	0.839045	N151	-0.3217	C201	-0.25027
C2	0.320067	C52	-0.76478	C102	0.26945	H152	0.095782	H202	0.152756
N3	-0.62106	C53	0.805714	C103	0.317303	H153	0.360937	C203	-0.25987
C4	-0.75759	C54	0.255863	H104	0.152998	C154	0.227088	H204	0.143953
H5	0.361481	C55	0.273634	C105	0.025462	H155	0.338023	C205	-0.25498
C6	0.79278	O56	-0.54675	H106	0.14424	C156	-0.2602	H206	0.167225
C7	0.845284	N57	-0.3252	O107	-0.55004	C157	-0.24973	C207	-0.13523
O8	-0.56288	H58	0.096679	N108	-0.31553	H158	0.167301	H208	0.180187
C9	-0.71567	N59	-0.31459	H109	0.091321	C159	-0.13478	C209	-0.14983
O10	-0.5512	H60	0.089399	N110	-0.61695	H160	0.180224	H210	0.166249
C11	-0.76876	C61	0.229803	H111	0.109791	C161	-0.14994	C211	0.231076
C12	0.804968	H62	0.340036	C112	0.021073	H162	0.15156	H212	0.181976
C13	0.251669	C63	0.224634	C113	0.227429	C163	0.023079	H213	0.151689
C14	0.274551	H64	0.324414	H114	0.324542	H164	0.145379	C214	0.022225
O15	-0.5475	C65	-0.26121	H115	0.360307	H165	0.090517	H215	0.14545
N16	-0.3219	C66	-0.25011	C116	-0.13771	C166	0.272895	N216	-0.32264
H17	0.097865	C67	-0.25776	C117	-0.14301	N167	-0.3154	C217	0.251534
N18	-0.31632	C68	-0.25311	C118	-0.25968	C168	-0.76504	H218	0.338759
H19	0.090022	H69	0.167434	C119	-0.25511	C169	0.224288	C219	-0.71354

C20	0.22964	C70	-0.13442	H120	0.152948	H170	0.325026	H220	0.097278
H21	0.337857	H71	0.179779	C121	-0.25773	C171	0.83799	C221	0.804618
C22	0.226195	C72	-0.14932	H122	0.142277	C172	0.807392	C222	0.787281
H23	0.325622	H73	0.168636	C123	-0.25875	C173	-0.25635	O223	-0.54753
C24	-0.26222	C74	-0.13716	H124	0.169246	C174	-0.25339	C224	-0.76694
C25	-0.2517	H75	0.18142	C125	-0.13556	O175	-0.54916	O225	-0.56157
C26	-0.25743	C76	-0.1476	H126	0.181927	C176	-0.74813	C226	-0.75293
C27	-0.25373	H77	0.151738	C127	-0.14579	O177	-0.54747	C227	0.842186
H28	0.168028	C78	0.021861	H128	0.167648	C178	-0.72211	C228	0.274264
C29	-0.13324	H79	0.145565	C129	0.229302	H179	0.168447	C229	0.319392
H30	0.180572	H80	0.151757	H130	0.183254	C180	-0.14042	O230	-0.55054
C31	-0.14811	C81	0.02477	H131	0.151047	H181	0.181451	N231	-0.31871
H32	0.168478	H82	0.143727	C132	0.023497	C182	-0.14666	H232	0.090394
C33	-0.13765	H83	0.096417	H133	0.143093	C183	0.791347	N233	-0.62083
H34	0.181445	C84	0.256312	N134	-0.31603	C184	0.311962	H234	0.109097
C35	-0.14747	N85	-0.32184	C135	0.269291	C185	0.256954	C235	0.227408
H36	0.150776	C86	-0.72187	H136	0.325112	H186	0.153207	H236	0.326809
C37	0.02206	C87	0.225929	C137	-0.75984	C187	0.026971	H237	0.361366
H38	0.144633	H88	0.338468	H138	0.09102	H188	0.142647	C238	-0.25852
H39	0.15196	C89	0.804708	C139	0.840062	O189	-0.56374	C239	-0.25349
C40	0.023903	C90	0.795259	C140	0.800899	N190	-0.6162	H240	0.168508
H41	0.143754	C91	-0.25841	O141	-0.55036	H191	0.111644	C241	-0.13689
N42	-0.61652	C92	-0.24994	C142	-0.75488	N192	-0.32305	H242	0.180923
C43	0.315555	O93	-0.54649	O143	-0.5453	H193	0.096298	C243	-0.14699
H44	0.359507	C94	-0.76033	C144	-0.71787	C194	0.019648	H244	0.152129
C45	-0.75195	O95	-0.56376	C145	0.792361	H195	0.359782	C245	0.023401
H46	0.110553	C96	-0.75561	C146	0.317437	C196	0.228424	H246	0.143968
C47	0.792645	H97	0.167042	C147	0.255926	H197	0.338984	H247	0.349873
C48	0.838619	C98	-0.13822	O148	-0.56304	C198	-0.13552	H248	0.349946
O49	-0.56402	H99	0.180317	N149	-0.61816	C199	-0.14555	H249	0.350113
C50	-0.72005	C100	-0.14891	H150	0.110051	C200	-0.25988	H250	0.351236
atom	charge								
H251	0.351067								
H252	0.350142								

Supplementary Table 4. RESP charge of [C_8mim] cation.

atom	charge	atom	charge
N1	0.181695	H20	0.030613
N2	0.191317	H21	0.030613
C3	-0.06863	C22	0.039193
H4	0.183748	H23	-0.01007
C5	-0.19128	H24	-0.01007
H6	0.232914	C25	0.016718
C7	-0.17416	H26	-0.00624
H8	0.238163	H27	-0.00624
C9	-0.36647	C28	-0.02099
H10	0.176588	H29	0.007561
H11	0.182566	H30	0.007561
H12	0.176588	C31	0.145127
C13	-0.1944	H32	-0.01848
H14	0.121881	H33	-0.01848
H15	0.121881	C34	-0.26599
C16	0.077533	H35	0.062954
H17	0.018682	H36	0.075142
H18	0.018682	H37	0.062954
C19	-0.04919		

Supplementary Table 5. RESP charge of [C_4mim] cation.

Atom	Charge	Atom	Charge
N1	0.233689	H14	0.121398
N2	0.173079	H15	0.121398
C3	-0.0903	C16	-0.00287
H4	0.19849	H17	0.032032
C5	-0.22014	H18	0.032032
H6	0.239756	C19	0.101092
C7	-0.1559	H20	0.021642
H8	0.233609	H21	0.021642
C9	-0.33135	C22	-0.28565
H10	0.169939	H23	0.085267
H11	0.169939	H24	0.085267
H12	0.169939	H25	0.085267
C13	-0.20926		

Supplementary Table 6. BCP data in atomic units for the noncovalent interactions within $[C_8mim]^+$ and TpBd COF.

$\rho(\mathbf{r})$	$\nabla^2\rho(\mathbf{r})$	$H(\mathbf{r})$	λ_1	λ_2	λ_3
0.0051	0.0148	0.0005	-0.0027	-0.0020	0.0195
0.0041	0.0115	0.0005	-0.0018	-0.0006	0.0140
0.0093	0.0220	0.0009	-0.0026	-0.0062	0.0308
0.0063	0.0244	0.0009	-0.0026	-0.0031	0.0301
0.0038	0.0134	0.0006	-0.0016	-0.0026	0.0176
0.0075	0.0212	0.0008	-0.0058	-0.0033	0.0303
0.0089	0.0285	0.0011	-0.0052	-0.0042	0.0379
0.0093	0.0220	0.0009	-0.0026	-0.0062	0.0308
0.0100	0.0301	0.0013	-0.0038	-0.0062	0.0400
0.0089	0.0295	0.0009	-0.0056	-0.0073	0.0424
0.0080	0.0280	0.0007	-0.0065	-0.0056	0.0402
0.0055	0.0163	0.0005	-0.0029	-0.0021	0.0213
0.0035	0.0112	0.0006	-0.0018	-0.0008	0.0138
0.0086	0.0239	0.0007	-0.0074	-0.0071	0.0384
0.0064	0.0161	0.0005	-0.0047	-0.0037	0.0245
0.0052	0.0139	0.0004	-0.0025	-0.0013	0.0177
0.0071	0.0186	0.0008	-0.0028	-0.0042	0.0257
0.0079	0.0226	0.0009	-0.0042	-0.0055	0.0324
0.0069	0.0179	0.0006	-0.0040	-0.0051	0.0270
0.0039	0.0103	0.0004	-0.0026	-0.0021	0.0150
0.0047	0.0155	0.0005	-0.0022	-0.0028	0.0205
0.0118	0.0293	0.0011	-0.0049	-0.0063	0.0404
0.0036	0.0096	0.0004	-0.0017	-0.0022	0.0135
0.0020	0.0066	0.0003	-0.0012	-0.0008	0.0087
0.0044	0.0119	0.0004	-0.0024	-0.0030	0.0173
0.0050	0.0135	0.0005	-0.0031	-0.0024	0.0190
0.0029	0.0096	0.0005	-0.0005	-0.0016	0.0117

Supplementary references

1. Chong, J.H., Sauer, M., Patrick, B.O., and MacLachlan, M.J. Highly stable keto-enamine salicylideneanilines. *Org. Lett.* **2003**, *5*, 3823-3826.
